# What the curtains do not shield: A phenomenological exploration of patient‐witnessed resuscitation in hospital. Part 2: Healthcare professionals' experiences

**DOI:** 10.1111/jan.15219

**Published:** 2022-03-24

**Authors:** Martina Fiori, Maureen Coombs, Ruth Endacott, Clara A. Cutello, Jos M. Latour

**Affiliations:** ^1^ Faculty of Health, School of Nursing and Midwifery University of Plymouth Plymouth UK; ^2^ School of Health and Social Care Edinburgh Napier University Edinburgh UK; ^3^ School of Nursing Midwifery and Health Practice Victoria University of Wellington Wellington New Zealand; ^4^ National Institute for Health Research London UK; ^5^ Faculty of Medicine, School of Nursing and Midwifery, Nursing and Health Sciences Monash University Frankston Victoria Australia; ^6^ Faculty of Health, School of Psychology University of Plymouth Plymouth UK; ^7^ Faculty of Business and Economics, Department of Marketing University of Antwerp Antwerp Belgium; ^8^ Faculty of Health Sciences, School of Nursing, Midwifery and Paramedicine Curtin University Perth Australia; ^9^ Hunan Children's Hospital Changsha China

**Keywords:** cardiac arrest, cardiopulmonary resuscitation, emergency treatment, health personnel, hospitals, interviews, nurses, patients, qualitative research, resuscitation

## Abstract

**Aims:**

To explore healthcare professionals' experiences of patient‐witnessed resuscitation in hospital.

**Design:**

Descriptive phenomenology.

**Methods:**

Healthcare professionals involved in hospital resuscitation activities were recruited from medical, intensive care, resuscitation and education departments in a university hospital in England. Data were collected through face‐to‐face and focus group interviews, between August 2018 and January 2019. Data were analysed using Giorgi's phenomenological approach.

**Results:**

Nine registered nurses, four healthcare assistants and seven doctors participated in four individual interviews and three focus groups. Findings were related to three themes: (1) Protecting patients from witnessing resuscitation: healthcare professionals used curtains to shield patients during resuscitation, but this was ineffective. Thus, they experienced challenges in explaining resuscitation events to the other patients and communicating sensitively. (2) Emotional impact of resuscitation: healthcare professionals recognized that witnessing resuscitation impacted patients, but they also felt emotionally affected from performing resuscitation and needed coping strategies and support. (3) Supporting patients who witnessed resuscitation: healthcare professionals recognized the importance of patients' well‐being, but they felt unable to provide effective and timely support while providing life‐saving care.

**Conclusion:**

Healthcare professionals involved in hospital resuscitation require specific support, guidance and education to care effectively for patients witnessing resuscitation. Improving communication, implementing regular debriefing for staff, and allocating a dedicated professional to support patients witnessing resuscitation must be addressed to improve clinical practice.

**Impact:**

The WATCH study uncovers patients' and healthcare professionals' experiences of patient‐witnessed resuscitation, a phenomenon still overlooked in nursing research and practice. The main findings highlight that, in common with patients, healthcare professionals are subject to the emotional impact of resuscitation events and encounter challenges in supporting patients who witness resuscitation. Embedding the recommendations from this research into clinical guidelines will impact the clinical practice of healthcare professionals involved in hospital resuscitation and the quality and timeliness of care delivered to patients.

## 1 INTRODUCTION

Sudden cardiac arrest is the third leading cause of death in Europe (Gräsner et al., 2021). Data on in‐hospital cardiac arrests are limited (Schluep et al., 2018) and mostly derived from the American Heart Association and the UK National Cardiac Arrest Audit (NCAA). In the United States, the annual estimated incidence is 9.7 in‐hospital cardiac arrests per 1000 admissions, with a survival rate of 25.8% (Holmberg et al., 2019), while in the United Kingdom the estimated incidence in 2019 was 1.0 per 1000 admissions, with a survival rate to hospital discharge of 23.5% (National Cardiac Arrest Audit, 2020). In Europe, the latest data from the European Resuscitation Council estimated the annual incidence of in‐hospital cardiac arrest between 1.5 and 2.8 per 1000 hospital admissions (Gräsner et al., 2021). These data indicate that it is possible for hospital patients to witness the cardiac arrest, and consequent resuscitation, of another patient.

Witnessed resuscitation in‐ and out‐of‐hospital has been investigated for decades and conceptualized in the literature, taking into account different environments and perspectives (Walker, 2006). Despite growing evidence on this topic, a gap in knowledge was identified around in‐hospital resuscitation witnessed by fellow patients (Fiori et al., 2017). Hence, the need for an exploratory study was identified to better understand the phenomenon of witnessed resuscitation in clinical settings from the perspectives of the patients who witnessed the event and the healthcare professionals (HCPs) involved in resuscitation activities (Fiori et al., 2019a). The WATCH (Witnessing an ATtempt of CPR in Hospital) study was therefore designed to answer the following research question: what are the experiences of patients and of HCPs regarding patients witnessing resuscitation of another patient in hospital? This paper reports on the HCPs' experiences, while findings from the experiences of patients who witnessed resuscitation are presented in the accompanying Part 1 paper (Fiori et al., 2022).

## 2 BACKGROUND

Healthcare professional perspectives of witnessed resuscitation in hospital have been studied in both critical care and non‐critical care settings, mostly in relation to family presence during resuscitation. Despite the endorsement of family‐witnessed resuscitation from international professional organizations (American Association of Critical‐Care Nurses, 2016; Australian and New Zealand Committee on Resuscitation, 2016; Emergency Nurses Association, 2018; Oczkowski et al., 2015) and the support confirmed in the 2021 European Resuscitation Guidelines (Mentzelopoulos et al., 2021), this practice still meets resistance from HCPs.

The main concerns for HCPs about family‐witnessed resuscitation relate to the risk for family members to be exposed to psychological trauma and stress, and the fear that the family could interfere with resuscitative efforts and affect team performances. HCPs have advocated guidance about decision‐making on family presence, logistics of conducting family‐witnessed resuscitation, and appropriate resource allocation to support resuscitation procedures in the presence of family members (Johnson, 2017; Sak‐Dankosky et al., 2014; Walker & Gavin, 2019).

While the debate on family presence has garnered support in recent years, other areas of witnessed resuscitation remain unexplored. This leaves clinical settings without policies and guidelines to optimize clinical practice. Patient‐witnessed resuscitation is one of these areas. While awareness and consensus around family‐witnessed resuscitation are gradually growing and HCPs are becoming more experienced in supporting family members during resuscitation of a relative, they might not be equally prepared to assist and support patients who witnessed a fellow patient undergoing resuscitation in hospital. Our study contributes to improving clinical practice, by uncovering the experiences of HCPs involved in resuscitation and in the care of patients who witnessed resuscitation in hospital wards.

## 3 THE STUDY

### 3.1 Aims

The aim of this study was to explore healthcare professionals' experiences of patient‐witnessed resuscitation in hospital and to identify the support they provide to patients who witnessed resuscitation.

### 3.2 Design

The study methodology, ethics and rigour are detailed in the published study protocol (Fiori et al., 2019a), and a summary is provided below. This qualitative study is reported following the consolidated criteria for reporting qualitative research checklist (Tong et al., 2007) (Appendix S2: COREQ checklist). A descriptive phenomenological approach was used to identify the experiences and meaning of patient‐witnessed resuscitation as perceived by HCPs in clinical practice (Giorgi, 2009).

### 3.3 Participants

A criterion‐based purposive sampling strategy was used to recruit participants from a single acute hospital in England, United Kingdom. Inclusion criteria were HCPs with >6 months of clinical experience who attended a resuscitation event in hospital in the last 6 months. To facilitate recruitment, the study was advertised through the hospital staff newsletter and presented to hospital department managers. HCPs interested in participating contacted the research team and, if eligible, received an invitation letter. Recruited participants received a participant information sheet and verbal explanation of the study. A total sample size of up to 20 participants for the face‐to‐face interviews and focus groups was considered sufficient to achieve data saturation (Braun & Clarke, 2013; Creswell & Creswell, 2018). According to the study protocol, one focus group was designed to capture the views of resuscitation team members separately from other HCPs, to avoid undue bias (Fiori et al., 2019a).

### 3.4 Data collection

Face‐to‐face individual and focus group interviews were conducted using an interview guide based on a few open questions to generate discussion. The interview guide was informed by a previously conducted Patient and Public Involvement (PPI) and stakeholder consultation (Fiori et al., 2019b) (Table 1). Individual and focus group interviews were conducted by the first author and audio‐recorded. In focus groups, the first author was supported by a second researcher (CAC) who documented visual cues and field notes. All individual and focus group interviews were conducted in a quiet room during participants' working time or study days, at the hospital or university site. All interviews and focus groups were audio‐recorded. Only the researchers and the participants were present during data collection. Data were collected between August 2018 and January 2019.

**TABLE 1:** Individual and focus group interview guide

Main question	I would like to know about your experience of CPR events that you attended in your ward. Would you like to share it with me?
Topics and prompts	Experiences Thinking about the last events, could you describe what happened during the CPR in your ward? Have you had any experience of a patient witnessing CPR on another patient?
	General attitudes What do you normally do when a patient in your ward witnesses CPR on a patient nearby? How do you approach the other patients on the ward when they witness CPR?
	Presence of policies What principles do you follow when you approach a patient who witnessed CPR on a fellow patient? Is there any policy in your ward to support patients exposed to CPR of another patient? What do you think of having guidelines to provide support to patients witnessing CPR?
	Needs, benefits and risks How do you think you could help patients witnessing CPR to cope with their experience? What other skills would be helpful for healthcare professional to support patients who witness CPR? In your opinion, what are the potential benefits and risks of providing support to patients after witnessing CPR?

### 3.5 Ethical considerations

Approval was gained on 2nd May 2018 by the National Health Service Health Research Authority (REC reference: 18/SW/0069; Protocol number: FHHS‐218744‐MF‐202; IRAS project ID: 218744) and on 18th May 2018 by the University Research Ethics Committee (FHHS‐218744‐MF‐202; Reference Number: 17/18–807). Study participants received verbal and written explanations before providing written consent. The audio‐recordings of the interviews and focus groups were destroyed after being transcribed and transcripts were anonymized. The personal information and confidentiality of participants were protected.

### 3.6 Data analysis

Individual and focus group interviews were transcribed and imported in QRS International NVivo v.12. Data analysis, following Giorgi's (2009) descriptive phenomenological approach, was conducted in accordance with the steps described in the study protocol (Fiori et al., 2019a). Core to this method is the description of the experiences from participants' point of view, the phenomenological reduction of raw data into phenomenological statements, and the search for the essential meanings of the investigated phenomenon. Two researchers (MF and CAC) independently coded all data, compared the coding process and developed an agreed coding framework, which was reiteratively reviewed by the senior researchers (JML, RE, MC). Themes and subthemes were developed inductively from the initial codes and reviewed in relation to the raw meaning units. The research team reviewed, discussed and agreed on the final findings (Supporting information: Coding framework extract).

### 3.7 Rigour

Trustworthiness principles for qualitative research were applied to maintain rigour throughout the study (Nowell et al., 2017). The researchers made efforts to bracket own past experiences and assumptions through self‐reflective writing and critical discussions with peer and senior researchers. Multiple data collection methods, namely individual and focus group interviews, were used to support triangulation. Congruence between two independent coders ensured confirmability. Findings provide comprehensive dataset representation and extracts of raw data for external assessment of interpretation.

At the time of the study, the first author was a postgraduate research student in nursing, three team members were senior academics in nursing and one team member was a postgraduate research student in psychology. All team members had training and previous experience in qualitative research. Participants were informed of the nursing and academic background of the first author when recruited and no previous relationship existed with them.

## 4 FINDINGS

Twenty HCPs involved in resuscitation in their clinical practice participated in the study. Fourteen participants worked in mixed medical specialities, including cardiology, gastrointestinal and liver services, four in the resuscitation and clinical education department, and two in acute and intensive care wards. Three focus groups (FG) were conducted: FG1 involved five junior doctors and one senior doctor; FG2 involved three healthcare assistants and three registered nurses; FG3 involved four nurses, members of the resuscitation team. Four participants, including three registered nurses and one senior doctor participated in individual face‐to‐face interviews. Focus groups lasted 34–69 min, while individual interviews 23–43 min. Table 2 summarizes the demographic and professional characteristics of the participants. Direct quotes of participants are presented using ID codes FG (Focus Groups) or Int (Interview) for focus group or individual interviews, and HCA (Health Care Assistant), RN (Registered Nurse), JD (Junior Doctor), SD (Senior Doctor), according to participants' profession.

**TABLE 2:** Participant demographic characteristics

Participant demographic characteristics	*n*
Gender	
Male	6
Female	14
Age (years)	
25–34	6
35–44	5
45–54	5
55–64	3
Not specified	1
Profession	
Healthcare Assistant (HCA)	4
Registered Nurse (RN)	9
Junior Doctor (JD)	5
Senior Doctor (SD)	2
Years of experience in that profession	
<10	9
10–20	7
>20	3
Not specified	1
Estimated number of CPR attended in whole career	
<10	3
10–20	5
20–30	4
>100	7
Not specified	1

Three themes and six subthemes were developed from the analysis of individual and focus group interviews. The essence of the participants' lived experience revealed that most HCPs considered resuscitation to be a stressful experience for both the patients who witnessed it and the staff who attended. HCPs attempted to protect patients from witnessing resuscitation, but available equipment, such as bed‐space curtains, offered limited effectiveness; hence HCPs faced challenges in communicating openly and sensitively with patients exposed to witnessing resuscitation events. HCP recognized that patients perceived an emotional impact from witnessing resuscitation, but they felt affected by attending resuscitation events too and needed coping strategies. Providing support to patients who witnessed resuscitation was considered at the core of the nursing role, although the prioritization of care towards resuscitation activities did not always make it feasible.

### 4.1 Protecting patients from witnessing CPR

This theme explored the challenges that HCPs experienced in protecting patients from witnessing resuscitation and in communicating effectively with them regarding the witnessed event. Most participants referred to bed‐space curtains as the principal means of protection of both the privacy of the patient undergoing resuscitation and the other patients in the room, despite acknowledging their limited effectiveness. All healthcare professionals were aware that the patients in the room had at least a partial understanding of the situation, therefore HCPs recognized their responsibility in communicating with them sensitively. While most participants agreed to provide patients with honest and truthful information, several HCPs felt limited in their practice by barriers such as lack of information on the patient after the cardiac arrest, fear of breaching confidentiality, and poor communication skills to hold sensitive conversations.

#### 4.1.1 Shielding patients behind curtains

Resuscitation events were described by participants as often hectic and stressful, with ‘*a pandemonium noise and people running, and curtains’* (FG3/RN20). Resuscitation team members explained that specific acts of HCPs, such as opening and closing the curtains around patients' beds, can represent for the other patients in the room ‘*obvious’* (FG3/RN20) indicators of a resuscitation event:

*“And then suddenly it goes quiet: all the screens stay around, and even worse when the screens are all open and that bed is swept off” (FG3/RN20)*.

Participants spoke of bed‐space curtains as the only screen available to protect the privacy of the patient undergoing resuscitation and to shield the other patients from witnessing a distressing event. However, most participants acknowledged the limited effectiveness of paper curtains in protecting the patients, and instead the risk of exacerbating their distress, by blocking them in their cubicles:

“Usually, we would pull a curtain around the other patients, which may make them feel quite blocked in, but that is all we can do to shield them. Unfortunately, because of the people and the equipment needed, they are not always shielded from it, we try our best, but we don't have anything else.” (Int1/HCA1).

Participants were aware that curtains could not block sounds of resuscitation activities. Some considered ‘*hearing’* as a ‘*particularly powerful sense’* (FG3/RN20). One nurse expressed the concern that hearing resuscitation is more distressing than watching it, as imagination may be ‘*even more frightening’* (FG2/RN12) than reality. Another nurse pointed out that curtains might give HCPs a false sense of protection, underestimating the impact on patients who overhear sounds and conversations around the resuscitation scene. Overall, participants agreed that despite HCPs' efforts in using bed‐space curtains, patients still ‘*hear everything, and they will realise that something is happening, just next to them’* (FG1/JD4). Resuscitation team members also recognized that patients' experience could be particularly distressing when they are exposed to the visual and auditory stress of witnessing the bereaved family of a deceased patient:

“RN19: It's not so much about cardiac arrest, once the family come in and they start crying.
RN18: The patients are going to know…
RN19: …then that sets the other patients off. So, if we just segregate it out and you just looked at the actual event, I think
  that's
  not as disturbing as when the relatives arrive. When they have heard screaming from relatives…”
  (FG3)

#### 4.1.2 Communicating with patients about CPR

Participants indicated that patients who witnessed resuscitation might already have an idea of what happened behind the curtains and of the outcome of the event, before the HCPs explained it to them. However, participants held different perspectives on disclosing information on the resuscitation event when patients posed questions. Most nursing staff appreciated that patients wished to understand what happened to their fellow patient, because ‘*they need closure’* (FG2/HCP10). Others instead, believed that patients are generally so ‘*frightened’* (FG1/SD7) that ‘*they don't want to talk to you’* (FG1/SD7). Senior professionals, both in nursing and medical roles remarked their responsibility of addressing patients' questions with ‘*honest and truthful’* (FG3/RN19) responses:

“If a patient says: ‘Did they make it?’ I tell them the truth. I say: ‘Unfortunately they didn't.’ And if they say to me: ‘We got really friendly over the last couple of days’ I just sit and say to them: ‘You know, they were very poorly, unfortunately, despite trying, they haven't made it.’ I think it's important we do tell them the truth.” (FG3/RN20).

Nevertheless, participants identified several challenges in communicating effectively with patients who witnessed resuscitation regarding the event. Ward nurses were often unable to answer patients' questions because they lacked information about the resuscitated patient, once moved from their ward. Uncertainties on ‘*breaching confidentiality’* (FG1/JD5) were identified as further barriers to speaking openly to patients. Importantly, some participants expressed difficulties in engaging in sensitive conversations involving death. A junior doctor voiced the concern of lacking expertise and confidence in talking to these patients about the resuscitation event and in handling emotions possibly arising from these conversations:

“Even if I speak to patients, I don't have the… I don't think I have enough expertise to talk with the emotions of the patients regarding this particular event honestly, so even if he's [patient] talking about it, I don't really know what…how should I handle it. I don't really know what to tell them and how to handle it.” (FG1/JD6).

Resuscitation team members confirmed these challenges and stressed the importance of HCPs' interpersonal and communication skills. They advocated that these skills need to be developed gradually, through ‘*exposure to emergency situations and critically ill patients’* (FG3/RN20). This would help HCPs build confidence about the nature of information that can be shared as well as awareness of the emotional impact that patients might experience and how to respond to it.

### 4.2 Feeling the impact of the CPR experience

This theme explored the perceptions of HCPs on patients' perceived impact of witnessing resuscitation, and on HCPs' own emotional reactions and needed support from attending resuscitation events. Healthcare professionals unanimously identified witnessing resuscitation as a stressful experience for patients. For most HCPs, patients tended to be more negatively affected by unsuccessful events and events involving fellow patients they have established a friendly relationship with during admission. However, although not directly investigated through the interview guide, participants commented extensively on their own perceived impact from attending resuscitation events, their emotional reactions and the need for coping strategies and support. Self‐care, informal peer support and structured debriefing were considered helpful strategies to maintain their well‐being after stressful events and be able to provide care and emotional support to the other patients.

#### 4.2.1 Recognizing emotional reactions in the patients

Participants identified witnessing resuscitation as a distressing experience for patients, defining it as ‘*upsetting*’ (FG2/RN11) and ‘*traumatizing*’ (Int4/RN16), with potential consequences such as post‐traumatic stress, ‘*particularly for people who understand the whole situation’* (Int2/SD2). Drawing from their experience, participants overall reported that patients' emotional reactions to witnessing resuscitation were influenced by its outcome. Healthcare assistants and nurses in FG2 shared the view that patients tend to feel reassured and grateful to the team when resuscitation is successful, but unsuccessful events could exacerbate their possible trauma:

“HCA10: I think, like you said, if somebody has a successful CPR and they're ok, after then the other patients stop and think: ‘We're in the right place’. They will say: ‘Oh, how efficient! Yeah, we're safe. We're in the right place… I'm not saying they think the opposite if somebody dies, but…if somebody dies, they just feel that shock and…”
RN9: So, I do think it's quite traumatic, particularly if the patient does not survive.” (FG2)

Most participants agreed that patients who spend longer periods in the same multi‐bedded room develop a special relationship, becoming ‘*comrades in arms’* or ‘*bay‐buddies’* (Int4/RN16). While in hospital, patients are each other's main support system: they share similar experiences as they undergo similar procedures. This could result in a close bond among them, described by some of the participants as *‘a real camaraderie, like “we're all in this together”’* (FG2/RN9). This rapport could be particularly significant when one of the patients suffered a cardiac arrest. In these cases, participants agreed that witnessing resuscitation would inevitably affect fellow patients and potentially cause them to fear that the same could happen to them:

“At the time when they're sick, that's their support network, you know, they've got ‘Mildred’ in the bed opposite and she's got the same thing that I've got and we're going for coffee afterwards and then she's just arrested and oh, she's passed away. I've lost a friend and that could happen to me and, yeah…” (Int3/RN15).

#### 4.2.2 Exploring emotional reactions and coping strategies of HCPs

Participants spoke of resuscitation events as stressful, not only for patients, but also for the HCPs involved. Their previous experience in managing cardiac arrests and resuscitation was considered crucial in determining the impact of the event on the HCPs. They were not always able to draw on confidence and knowledge, especially junior staff, who might find unexpected emergency situations overwhelming. Participants described coping mechanisms adopted to process resuscitation experiences, such as ‘*compartmentalizing’* (Int2/SD2) and establishing a ‘*professional distance’* (Int4/RN16), as strategies to ‘*stop* [themselves] *from falling apart’* (Int1/HCA1). Other participants instead, highlighted the difficulty in distancing themselves from the resuscitation event:

“Yes, you could detach […] But then as soon as the family comes in, then it's like ‘Oh God, they are humans!’ That's the bit that gets me: they belong to somebody. And all kind of go at that point.” (FG2/HCA14).

Nurses remarked that awareness of the emotional impact of resuscitation experiences on HCPs is essential to help staff cope with their own reactions and, most importantly, to enable them to support their patients. Participants recognized the importance of promoting an effective practice of self‐care, such as *“making sure you speak to colleagues, that you get the support from your network in your own way, that you are rested and nourished* […] *so that you can be balanced to make the best clinical decisions”* (Int2/SD2); and implementing team coping strategies after stressful events:

“I feel really strongly that as, like, healthcare staff we need to look after ourselves to look after our patients and I think by doing, like, a structured debrief or even an unstructured debrief it would help the staff process what happened, acknowledge it and be able to move on and get back to looking after patients.” (Int4/RN16).

Participants described informal peer support as a strategy to check closely on the team members and provide reassurance to less experienced members of staff. For some, colleagues are “*the best support”* (Int1/HCA1), because they are “*the people that were there with you, who took part in that with you”* (Int1/HCA1) and are a unique source of understanding and trust. Formal debriefing was also recognized as a valuable method to reflect on and process the resuscitation event. Regular debriefing was considered by participants to provide an opportunity to analyse “*technical aspects”* (Int4/RN16) of the HCPs response, but also an important tool to look at the *“wellbeing of the team”* (FG1/SD7). However, most participants voiced a lack of guidance and lack of a standardized approach to implement effective debriefing practice consistently after resuscitation events.

### 4.3 Supporting patients who witnessed CPR

This theme explored HCPs' support practice to patients who witnessed resuscitation and the barriers to patient support while prioritizing life‐saving care during resuscitation. HCPs identified the provision of emotional support to the patients who witnessed resuscitation at the core of nursing practice. Nurses also advocated the role of dedicated staff to look after the other patients during resuscitation. Some participants recognized examples of effective practice, such as a previously implemented counselling service for inpatients and the role of the chaplaincy, while others advocated clearer information on what support services are available to patients after the event. Participants also highlighted the challenges in taking care of other patients while focusing on the management of the cardiac arrest, and in offering adequate time for emotional support after resuscitation while facing time pressures and limited staffing.

#### 4.3.1 Caring for the well‐being of patients witnessing CPR

Participants affirmed that looking after the emotional well‐being of patients after witnessing resuscitation is at the core of HCPs' duty. Both nurses and doctors identified nursing staff as best suited overall to offer emotional support to patients, because they *“know those patients better than any doctoring staff.”* (FG1/JD8). Nevertheless, senior doctors also acknowledged their responsibility of offering the patients the time and opportunity to talk to a member of staff and be reassured:

“I thought we have to offer the time and options and just say: ‘You have been involved in something potentially quite traumatic and these are the options: you can contact your GP, or this is the service we provide you, or would you like to talk through what's happened or any questions you've got, and you want me to answer anything?’ Because often it is just information that they need.” (Int2/SD2).

Nursing staff, however, identified shortfalls in providing support at the time of the event, and in signposting patients to specific services after resuscitation. Some nurses brought the examples of previous successful initiatives, such as “*a trained nurse counsellor”* (FG2/RN9) in specific departments but stressed the lack of established support systems for patients across clinical wards. In the absence of these, the pastoral and spiritual care service of the hospital was referred to as the only resource available to assist nursing staff in supporting patients who witnessed resuscitation. To improve current practice during resuscitation events, one participant suggested having a dedicated staff member who takes care of the other patients:

“You have the student nurse or HCA or a nurse that's come over from another ward to help out. You can have something hanging on the trolley to give to someone and say: ‘You are in charge of everybody else’. They've got that on, and everyone knows not to ask them to get anything because they're looking after everybody else. It makes everybody else aware of everybody else as well.” (Int3/RN15).

Resuscitation team members in FG3 reinforced the need to improve current practice and reflected on their educational role during HCP training sessions in raising staff's awareness of patients who witness resuscitation events and of the support available to them.

#### 4.3.2 Prioritizing care during CPR

Participants discussed the challenges in taking care of the other patients during resuscitation. Most HCPs shared the view that, during the event, the entire workforce is focused on the patient suffering the cardiac arrest. Hence, the delivery of resuscitation care has the priority; the attention and support immediately available for the surrounding patients might be limited. In certain cases, participants were aware of these challenges, and expressed concerns that although the other patients were safe, because they ‘*are breathing, they're actually okay’* (Int4/RN16), they might be overlooked, ‘*sat behind the curtains and sort of looking around maybe on their phone, just left to their own devices*’ (Int3/RN15). Other participants instead, stressed that during resuscitation events, they are immersed in their task, focusing exclusively in responding to the cardiac arrest and performing quality resuscitation, so that they are unable to dedicate attention also to the other patients in the room:

“I am thinking about what happens during the CPR, and on the process itself… you need to focus on the patient, who's actually dying or dead, but not… the surroundings. (…) That's what I am thinking about, but honestly, I don't really think about… I've never thought about the other patients surrounding it.” (FG1/JD6).

After resuscitation, time pressures and short staffing levels were further challenges discussed by participants. Most junior doctors agreed that ‘*for someone who witness it* [CPR]*, the ground reality is the staff themselves don't have time to actually counsel them’*. (FG1/JD3). Doctors responding to emergency calls were usually unable to visit the patients who witnessed resuscitation in the room, due to the pressure of their workload. Nurses and healthcare assistants commented particularly around staff shortages, which could affect the provision of sufficient support to patients who witnessed resuscitation. These issues were also echoed by the resuscitation team nurses, who supported their colleagues in clinical wards:

“RN19: They [ward nurses] have not got time to actually go [and ask]: ‘Is there anything you want to talk about?
RN17: It's the physical issue though, that takes over, that becomes the priority really, the drug rounds, dressing, the list of things that have to be done and the psychological support probably takes a backseat, which is a shame really.” (FG3)

### 4.4 Synthesis of the experiences of patients and HCPs

**FIGURE 1:**
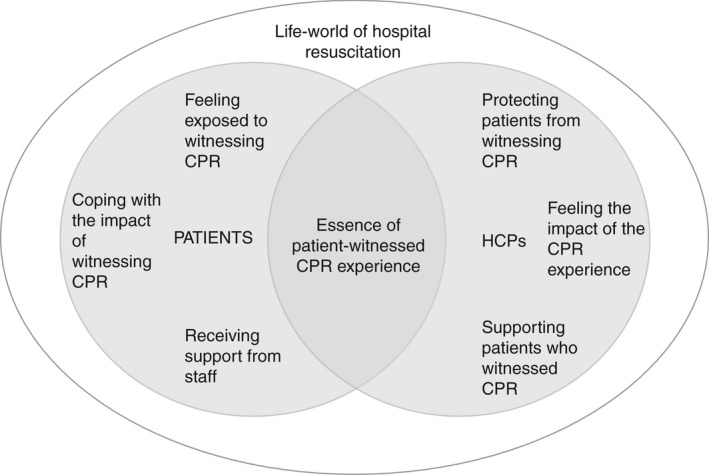
Experiences of patient‐witnessed resuscitation for patients and healthcare professionals

The experiences of patients who witnessed resuscitation and of HCPs, explored in the WATCH study and reported in Part 1 (Fiori et al., 2022) and in this Part 2 paper, are brought together to summarize the common meaning of patient‐witnessed resuscitation, within the hospital life‐world (Figure 1). Patients and HCPs had a shared rational understanding of hospital resuscitation: they were aware that emergencies and fatalities are part of hospital life and resuscitation is a potential consequence of such events. Despite so, the phenomenon of witnessing resuscitation was loaded with emotional significance. The inefficacy of bed‐space curtains to protect patients from witnessing resuscitation was recognized by both patients and HCPs; therefore, patients felt exposed to a distressing event, and HCPs felt unable to shield them. While HCPs worried that the outcome of resuscitation would impact on patients' witnessing experience, patients found reassurance in observing HCPs' response to cardiac arrest, with a consequent sense of restored safety and increased confidence in the healthcare staff. Both patients and HCPs had negative emotions associated with the resuscitation events and expressed the need of coping strategies to process the experience. For patients, witnessing resuscitation of a patient they knew, and witnessing the reactions of the family of the victim, proved particularly difficult. HCPs expressed the need for debriefing space for them to provide better support to patients. Emotional support for patients after witnessing resuscitation events was identified as beneficial by patients and HCPs, although its efficient delivery was hindered by a lack of resources and of clear guidance on available support pathways for patients.

## 5 DISCUSSION

The findings reported in this paper provide insight into the experiences of HCPs around patient‐witnessed resuscitation in hospital and help understand the challenges in patient support. The theme of protecting patients from witnessing resuscitation emerged as a key aspect of HCPs' experience. Bed‐space curtains were identified as ineffective measures, failing to protect patients from witnessing a stressful event and potentially worsening their distress. These findings are congruent with those from patient interviews conducted as part of the WATCH study (Fiori et al., 2022), with patients feeling only partially protected when witnessing resuscitation and stuck in their bed‐space. Participants also remarked that curtains were ineffective in protecting both the visual and auditory privacy of the patient undergoing resuscitation, posing ethical questions for clinical practice. Similarly, during emergency procedures, conversations and patient's information might be overheard by nearby patients, with implications for patient satisfaction and experience (Lin et al., 2013). In the United Kingdom, the duty of confidentiality during resuscitation is reinforced in national guidance (British Medical Association et al., 2016). However, no specific regulations exist for HCPs in the context of resuscitation in multi‐bed rooms. Since the other patients in the room might be aware of resuscitation and of its outcome, HCPs face uncertainties around the ethical implications of addressing patients' questions about the witnessed event. Awareness that HCPs might be witnessed by patients while performing resuscitation and clearer guidance on what information HCPs can disclose can support them in tackling communication challenges with these patients.

HCPs identified a lack of confidence and expertise in holding conversations about witnessed resuscitation events as further barriers to effective communication with patients. Previous studies suggest that these barriers constitute an issue in most resuscitation discussions. A literature review identified a lack of communication skills, low confidence, inexperience and discomfort, in both doctors and nurses who have resuscitation conversations with patients (Mockford et al., 2015). It can be argued that HCPs might avoid difficult conversations because they want to protect patients from distressing discussions and strong emotional reactions. Similarly, HCPs might also fear their own reactions or not feel confident in managing patients' emotions (Hurst et al., 2013). Nevertheless, the importance of improving communication skills to support patients who witnessed resuscitation was recognized by HCPs in the study, and the lack thereof was a concern for several of them. This was also reflected in patient interviews (Fiori et al., 2022). A meaningful conversation with an HCP after witnessing resuscitation can be a valuable opportunity not only to alleviate patients' distress, but also to discuss patients' concerns around resuscitation and their own decisions, and as such should be encouraged in clinical practice.

Participants recognized witnessing resuscitation as a distressing experience for patients, especially when involving a fellow patient they felt connected to. HCPs' views reflected those of the patients interviewed in the WATCH study (Fiori et al., 2022). Similar findings were identified in other settings. For instance Andersen et al. (2015) argued that the complexity of the relationships developed among oncology patients might influence their experience both positively and negatively. Therefore, awareness of patients' social dynamics in hospital rooms, especially in distressing situations such as witnessing resuscitation, can give HCPs insight on patients' emotional impact, and gauge the support they might need. Participants also identified that witnessing resuscitation has a more negative impact on patients when unsuccessful. In this case, findings differ from those of patients' interviews, where patients' emotional impact did not appear to vary in relation to the resuscitation outcome, but rather to the stage of the cardiac arrest response. Patients' initial negative emotions were often followed by a positive sense of reassurance after observing HCPs resuscitation efforts (Fiori et al., 2022). However, this aspect did not emerge from HCPs' data, suggesting that they might not be aware of such an outcome in patients' witnessing experience. Sharing to HCPs that patients could feel reassured from witnessing their response to a cardiac arrest is valuable. This could help HCPs manage their own negative feelings following unsuccessful resuscitation and feel more prepared to support the patients who witnessed it.

The emotional impact that HCPs perceived from performing resuscitation emerged as an element that can affect their ability to provide effective support to patients who witnessed resuscitation. Junior staff, in particular, were identified as more likely to be negatively affected by resuscitation events and to require more support, consistently with previous literature (Ranse & Arbon, 2008). Individual coping strategies, such as self‐care, informal peer support and structured debriefing were found beneficial for HCPs to process their experience and feel emotionally fit to offer support to patients who witnessed resuscitation. Debriefing with HCPs after critical incidents have been increasingly valued in multiple clinical settings, as it can contribute to support staff's well‐being and to maintain a healthy work environment, which in turn benefits patients' care (Couper et al., 2013). However, in our study, this was poorly used in practice. Its rare application suggests the need of organizational and educational interventions to promote a regular implementation, with benefits for both HCPs' and patients' well‐being.

Providing support to witnessing patients was considered a core aspect of HCPs' role, but some participants expressed difficulties addressing patients' emotional needs. Drawing attention to the other patients during a cardiac arrest response could be challenging, but it is crucial that HCPs develop an understanding of the distress patients might experience in witnessing resuscitation. Further identified limitations to patient support were time pressures and limited staff. In these circumstances, the hospital chaplaincy service was considered by study participants as a valuable resource for distressed patients. Hospital chaplains have been historically involved in patient emotional support and their role in family‐witnessed resuscitation events has been documented in literature (James et al., 2011). New empirical research is also supporting the role of social workers as a family support person during hospital resuscitation (Firn et al., 2017). The suggestion of a dedicated professional to support patients witnessing resuscitation, could have positive implications in clinical practice, as previously discussed in Part 1 (Fiori et al., 2022). While in our study nurses were identified as best placed to look after patients witnessing resuscitation, it is worth considering whether a multi‐disciplinary approach could benefit patient experience. This could include chaplains and social workers, without further weighing on nurses' workload pressures.

Finally, it remains unclear whether study participants were aware of other structured support pathways for patients currently available from the hospital. The role of the resuscitation and education department in this sense appears crucial to identify such opportunities and educate HCPs so that they can support patients who witnessed resuscitation consistently across clinical settings. Further research exploring the perspectives of HCPs in other clinical settings, and of other healthcare professions and support workers involved in hospital cardiac arrests would contribute to understand the phenomenon of patient‐witnessed resuscitation.

### 5.1 Limitations

This qualitative study has provided context‐specific findings, which might have limited generalisability in other settings or populations. Sample bias needs to be considered: participants only included nurses, healthcare assistants and doctors who voluntarily chose to participate in individual and focus group interviews, therefore potentially representing partial views of the population. The three focus groups were conducted with single‐profession participants: while conducting a separate focus group with members of the resuscitation department was deliberate, due to their leadership role, composition of the other focus groups was informed by pragmatic and organizational reasons. Therefore, although interaction between different levels of expertise within the same profession was demonstrated, the interaction between nursing and medical professions was not achieved. Potential bias due to different rank and seniority in single profession focus groups should also be acknowledged. Nevertheless, findings are strengthened by the combined use of individual and focus group interviews, which proved to be a successful method for data collection and helped to illuminate the understanding of participants' experiences.

## 6 CONCLUSION

Findings of this study help understand the perspectives of HCPs around the phenomenon of patient‐witnessed resuscitation, contributing to identifying challenges and limitations in HCP practice and in supporting patients who witness resuscitation. Multiple solutions have been identified in this study to improve clinical practice, including, but not limited to, the presence of a dedicated support person for patients witnessing resuscitation events, further reinforced by the hospital chaplains and a multi‐disciplinary approach when available. Simulation training with standardized actors might represent a valuable solution to meet HCPs' educational needs in developing communication skills around resuscitation conversations with patients. Finally, implementing effective debriefing and support practices for staff attending resuscitation will be beneficial for HCPs' well‐being and have an impact on the quality of support they can offer to patients witnessing resuscitation.

## CONFLICT OF INTEREST

No conflict of interest has been declared by the authors.

## AUTHOR CONTRIBUTIONS

All authors have agreed on the final version and meet at least one of the following criteria (recommended by the ICMJE*): 1) substantial contributions to conception and design, acquisition of data, or analysis and interpretation of data; 2) drafting the article or revising it critically for important intellectual content. * http://www.icmje.org/recommendations/

### PEER REVIEW

The peer review history for this article is available at https://publons.com/publon/10.1111/jan.15219.

## Supporting information


Appendix
