# Supplementary material for: What the curtains do not shield: A phenomenological exploration of patient‐witnessed resuscitation in hospital. Part 2: Healthcare professionals' experiences
Source: J Adv Nurs. 2022 Mar 24;78(8):2596–607. doi: 10.1111/jan.15219 (PMC9544000; doi:10.1111/jan.15219)
Supplement: Supplementary file 1 — Appendix [file JAN-78-2596-s001.docx]

**Supporting information: Coding framework extract**

The following table illustrates the process of data analysis following Giorgi’s phenomenological method (Giorgi, 2009) and the development of the theme “Protecting patients from witnessing CPR”.

**Theme 1: Protecting patients from witnessing CPR**

Subtheme 1: Shielding witnessing patients behind curtains

| **Meaning units (Raw Data)** | **Phenomenological Statements** | **Nvivo Codes** |
| --- | --- | --- |
| “Usually, we would pull a curtain around the other patients, which may make feel them quite blocked in, but that is all we can do to shield them. Unfortunately, because of the people and the equipment needed, they are not always shielded from it, we try our best, but we don't have anything else.” (Int1/HCA1) | HCA1 reflects on their practice in protecting other patients when a resuscitation event occurs in a multi-bedded room. HCA1 describes their attempts in shielding the other patients using the curtains around the bed but recognises that these are often insufficient to provide protection and might also have the counter effect of blocking patients in their cubicle. | **Drawing the curtains** |
| "Everybody that I have seen has been very good at making sure that the curtains in the beds and surrounding areas are shut, you know, which is really good for the privacy of the patient who's having CPR because it's really difficult to keep that curtain shut because you've got so much equipment that they're kind of just taking up half the bay, so they are very good at shutting the curtains or putting up screens for the rest of the patients. but then the curtains are shut and the patients are just left behind the curtain by themselves listening to everything that's going on." (Int3/RN15) | RN15 describes the professionalism of the team members in drawing the curtains around the beds during resuscitation attempts. RN1 reflects on the benefits of this practice in protecting the privacy of the cardiac arrest victim, but is also concerned about the other patients, who are left behind the curtains, still aware of the surrounding situation. |  |
| "Sometimes I wonder if actually hearing something happening is almost worse than seeing it as well because I think the imagination of what it is and you don't know you... just hear these counting and lots of noise and machines and shocks, and machine and stuff like that. It's ...and obviously it's not... it's not something that they should see but I thought I should imagine it's even more frightening they can hear just everything." (FG2/RN12) | RN12 reflects on the impact on patients of hearing resuscitation of another patient across the curtains and questions whether hearing the noises and sounds of machinery could be more frightening than seeing real images and have a realistic understanding of resuscitation. | **Patients hearing CPR** |
| "I know we [HCPs] don't consciously think that they [curtains] are sound blockers, but sometimes the way people go on you feel like they don't really appreciate that noise is actually travelling past curtains. So they might hear some aspects of the patient's history or they might hear what's going on or even like one of the most traumatising things you can see, that I've seen, is like intraosseous access, where they drill into someone's bone and imagine being at the other side of the curtain and hear this drill going on." (Int4/RN16) | RN16 reflects on the practice of HCPs during resuscitation events and explains how there is a general tendency to overestimate the sound blocking properties of bed-space curtains. As a consequence, RN16 explains that patients might overhear conversations regarding the patient undergoing resuscitation, or parts of treatments and procedures such as an intraosseous access. RN16 reflects on the potentially traumatic impact that overhearing resuscitation could have on patients. |  |
| "RN11: It was just me and you, doing sort of chest compressions and airbag wasn't it? HCA10: Yeah. RN11: But there was nobody to actually go around and check on the other patients. HCA10: No, no... for quite a while... RN11: Because it took quite a long time for the crash team to arrive... HCA10: So what they actually did witness was doing chest compressions. RN11: and with this gentleman that we are talking about we’ve gotten him back four times. HCA10: Yeah, it went on for... RN11: it went on for a long time, and eventually he went, but for them [other patients], they must have thought you know, he's breathing, he's fine, Okay, and then we're back on his chest again, you know it is sort of... HCA10: Yeah. RN11: And it was quite a traumatic one, you can understand why they were all upset. HCA10: And then the man's family came in, his daughter came in and obviously she was really upset and the three other patients witnessed all that as well. It's you know... the relatives..." (FG2) | RN11 and HCA10 recall a resuscitation attempt they attended together in a multi-bedded room. They described that during the initial response RN11 and HCA10 were the only members of staff present and were occupied in providing chest compressions and mask ventilation.  RN11 and HCA10 recalled that the patient was resuscitated four times over a long period of time, whilst the other patients in the room could witness the whole situation, as no one was available at that time to close the curtains around them. RN11 and HCA10 reflected on the emotional impact of this event on the other patients, as they perceived the other patients to be upset during and following the event. Patients' emotions were also influenced by witnessing the visit of the victim's family, who were distressed by the event. | **Patients seeing CPR** |
| "I've had patient when I was a thoracic nurse, a patient who arrested right in the middle of the bay and it was a pretty awful arrest and there was lots of witnesses of the patients so they witnessed quite a bit until...because I had to keep going whilst everybody else was running around the screen and there was no screen across or anything." (FG3/RN19) | RN19 described her past experience of responding to a particular cardiac arrest and performing CPR in a multi-bedded room. RN19 recalled being in the middle of the room, initially unable to close the curtains around the bed, alike the other members of the team occupied in the immediate response. RN19 explained that in that occasion, the other patients were not protected from eye-witnessing CPR. |  |

Subtheme 2: Communicating with witnessing patients about CPR

| **Meaning units (Raw Data)** | **Phenomenological Transformation** | **Nvivo Codes** |
| --- | --- | --- |
| "Like I said, there's no closure for them, is there? They are just hanging on, don't they? This person was unwell and has been taken off somewhere. Is he still alive? This, you know the balance of wanting to know that, but sometimes we don't even know that because they get moved on and then we don't know what happens to them. So… you can't tell them because you don't know yourself." (FG2/HCA10) | HCA10 discusses patients' need of closure after witnessing a resuscitation attempt on another patient. HCA10 understands patients' need of information, but feels unable to answer their questions because also HCPs often lack information about the cardiac arrest victim. | **Witnessing patients need information** |
| "I don't know what is the right thing to do either, because uhm... I am not sure we can tell too much to other patients, for a matter of confidentiality you can't start explaining every patient and go through what was happening because I think that's not right neither." (FG1/JD5) | JD5 explains their challenges in communicating with witnessing patients after resuscitation events for the uncertainties in breaching confidentiality when disclosing information with them. | **Confidentiality** |
| "It's very difficult [to talk to witnessing patients] without crossing the confidentiality boundaries for what you can do. I don't really know what to suggest that will be good without going too far on confidentiality, because you know, we can't really say what happened to the patients or why or what the outcome is. I do feel they witness this horrible event that isn't what they thought it was going to be and then there is nothing." (Int1/HCA1) | HCA1 expresses their uncertainties in talking to witnessing patients and answering their questions without breaching confidentiality. HCA1 is aware that the support that can be offered by HCPs to witnessing patients might be limited by confidentiality boundaries and it might not be sufficient for patients. |  |
| "And there is something else to add. Even if I speak to patients I don't have the… I don't think I have enough expertise to talk with the emotions of the patients regarding this particular event honestly, so even if he (patient)'s talking about it I don't really know what...how should I handle it. So that's another thing. It never happened, but I'm assuming that this is the next level they [HCPs] don't really know what to tell them and how to handle it." (FG1/JD6) | JD6 explains that they feel unskilled to talk with witnessing patients about the resuscitation event and about their emotions arising from the experience. JD6 explains that they would not know how to manage patients emotions, and speculates that other HCPs might feel the same. | **Expertise in communicating with patients** |
| "It would be about confidence and knowledge and understanding. and we will have buckets of that from a very long time being exposed to very critically unwell patients because of the type of careers we've all moved in. And also dealing regularly with medical emergencies and cardiac arrest. So we've, like we've seen hundreds, most of the staff that are in this hospital if you ask how many cardiac arrests they've seen, it is maybe one or two and some people none in the whole of their career. So actually that's very difficult..." (FG3/RN19) | RN19 explains how the expertise in talking to patients is developed through confidence, knowledge and understanding and that resuscitation officers have developed this expertise through prolonged exposure to critical situations and critically ill patients. RN19 however, is aware not all HCPs have the same clinical background, and most of them have a limited exposure to critical situation, and therefore limited expertise in dealing with critical situations and communicating with patients. |  |
| RN19: We have to be truthful, we have to be honest. And it's just learning, and developing those communication skills. And communication is a very difficult thing to learn isn't it? Until you've got the experience to be able to pass that information across. RN20: It's communication, it's breaking bad news. It's all those skills that you get taught as a healthcare professional. I'm not saying they teach us every element and exposure, experience... RN19: exposure is going to be huge! RN20: Yeah, but I think it's how they [HCPs] perceive the conversation themselves, unfortunately. That they anticipate it's going to be hard because what you're actually going to do is sit down and say you know: "How are you? I realize you've just witnessed something. So are you okay? Can I get you a cup of tea?" You know, anything! It's the simpler things, isn't it? RN17: Because I can put myself back 28 years ago, I wouldn't have that confidence to ask something like that. | RN19, RN20 and RN17 discuss on the importance of developing communication skills to be able to communicate sensitive information to patients and breaking bad news.  RN20 reflects on the fact that one important factor in carrying out difficult conversations with patients is how the HCPs perceive that conversation for themselves. RN20 explains that HCPs perceive conversation with patients will be difficult because they lack the communication skills to be able to interact effectively with patients and offer them basic emotional support.  RN17 remarks that confidence in handling difficult conversations is developed through long-term experience. | **Lack of communication skills** |
